# PC-PVT: A platform for psychomotor vigilance task testing, analysis, and prediction

**DOI:** 10.3758/s13428-013-0339-9

**Published:** 2013-05-25

**Authors:** Maxim Y. Khitrov, Srinivas Laxminarayan, David Thorsley, Sridhar Ramakrishnan, Srinivasan Rajaraman, Nancy J. Wesensten, Jaques Reifman

**Affiliations:** 1 Biotechnology High-Performance Computing Software Applications Institute (BHSAI), Telemedicine and Advanced Technology Research Center (TATRC), U.S. Army Medical Research and Materiel Command (USAMRMC), Attn: MCMR-TT, 504 Scott Street, Fort Detrick, MD 21702 USA; 2 Behavioral Biology Branch, Center for Military Psychiatry and Neuroscience, Walter Reed Army Institute of Research, 503 Robert Grant Avenue, Silver Spring, MD 20910 USA

**Keywords:** Psychomotor vigilance task, Reaction time, Data collection, Performance prediction

## Abstract

Using a personal computer (PC) for simple visual reaction time testing is advantageous because of the relatively low hardware cost, user familiarity, and the relative ease of software development for specific neurobehavioral testing protocols. However, general-purpose computers are not designed with the millisecond-level accuracy of operation required for such applications. Software that does not control for the various sources of delay may return reaction time values that are substantially different from the true reaction times. We have developed and characterized a freely available system for PC-based simple visual reaction time testing that is analogous to the widely used psychomotor vigilance task (PVT). In addition, we have integrated individualized prediction algorithms for near-real-time neurobehavioral performance prediction. We characterized the precision and accuracy with which the system as a whole measures reaction times on a wide range of computer hardware configurations, comparing its performance with that of the “gold standard” PVT-192 device. We showed that the system is capable of measuring reaction times with an average delay of less than 10 ms, a margin of error that is comparable to that of the gold standard. The most critical aspect of hardware selection is the type of mouse used for response detection, with gaming mice showing a significant advantage over standard ones. The software is free to download from http://bhsai.org/downloads/pc-pvt/.

A review of the current literature concerning reaction time (RT) testing suggests that achieving the necessary precision and accuracy in collected data (typically on the order of a few milliseconds) requires the use of specialized hardware (see Li, Liang, Kleiner, & Lu, 2010; Ohyanagi & Sengoku, 2010). A commercially available, off-the-shelf (COTS) personal computer (PC) is an obvious first choice for such a testing platform, due to its wide availability and relatively low cost. However, a COTS PC is considered to be an unreliable device for RT measurements because of its inherent, seemingly uncontrollable, delays. Indeed, computer monitors, input devices, and software are not designed with millisecond-level accuracy of operation in mind, primarily because such time scales are not required, or even noticeable, in general-purpose computing. Most currently available monitors refresh their screens at 60–120 Hz, universal serial bus (USB) keyboards and mice are typically polled at 125 Hz, and additional delays are introduced by the hardware design, operating system (OS), device drivers, and other background processes. Without any special consideration, these sources of delay may add up to an overall timing error on the order of 100 ms with substantial variability, which is unacceptable for most neurobehavioral performance studies, in which a small change in RTs may be of operational or clinical relevance.

The typical solution to this problem has involved the development of hardware platforms that are dedicated to the single task of measuring simple visual RT (Dinges & Powell, 1985; Li et al., 2010; Repperger, Jacobson, Walbroehl, Michel, & Goodyear, 1985; Thorne et al., 2005). Some of these platforms are standalone, containing their own methods for stimulus presentation, response detection, and calculation of the stimulus–response interval via an internal timer. Others use the PC for certain functions (such as stimulus presentation), but still provide their own timing mechanisms that are free from the aforementioned errors. The obvious disadvantage to this approach is the requirement to design/purchase, configure, and maintain additional dedicated hardware for what is otherwise a fairly simple task. All such devices provide their own methods of recording RT data and transferring the results to a PC for analysis, but these steps frequently involve manual data transfer and organization, procedures that increase the likelihood of data loss or corruption due to human error. Furthermore, the lack of consistency among output formats means that additional work is generally required to convert and arrange the results before analyses can be performed.

One such standalone device is the PVT-192 (Ambulatory Monitoring, Inc., Ardsley, NY; Dinges & Powell, 1985), which is considered the “gold standard” for simple visual RT testing (the PVT-192 also produces 1000-Hz tones that are accessible via a headphone jack; this audio capability was not evaluated, nor will it be described further here). In an exemplar study, each subject performs 5- or 10-min RT sessions spaced at predetermined intervals (e.g., every 2 h) for several days or weeks, where each session consists of 50 to 100 stimulus–response events. The stimulus consists of a four-digit millisecond counter that appears in the light-emitting diode (LED) dot-matrix display, and the response consists of a left or right button press, depending on the configuration. The time difference between the stimulus presentation and the response constitute the subject’s RT. Each RT value is stored in the device and then uploaded to a PC, where the individual RTs are postprocessed with the REACT (Ambulatory Monitoring, Inc., Ardsley, NY), or other commercially available software, into summary statistics, such as the mean RT or the number of lapses (RTs > 500 ms) per session.

We ported the features of the PVT-192 to the PC in order to realize a number of benefits. First, the PC has an advantage over a custom hardware platform in terms of ease of use, availability, support, cost, and user familiarity. Second, the testing and analysis modules can be integrated into a single package, which allows us to bundle more advanced analysis tools, such as our real-time individualized performance prediction algorithms (Rajaraman, Gribok, Wesensten, Balkin, & Reifman, 2008, 2009). Integrating such functionality into a specialized hardware system would be difficult due to a lack of sufficient processing power and memory. By using the PC, we can update the performance predictions as soon as the current session is finished, and these predictions are immediately made available to the investigators. The software is written in a way that makes it relatively simple to include other analysis methods in the package, enabling side-by-side comparison of different algorithms and summary statistics. Finally, the storage, organization, and data export tasks are simplified by removing the need for manual data manipulation and uploading from the specialized hardware to a computer. The software automatically organizes data into separate folders according to study name and subject identifiers, which should help minimize the potential for data loss, and makes the raw and processed data available for export in comma-separated value (CSV) format. In environments where multiple networked computers are used for testing, the PVT sessions and analysis results can be automatically saved to a shared network drive, creating a central data repository.

Realization of these benefits depends on the accuracy and precision with which our PC-PVT software measures RTs. Because the PVT-192 is a specialized real-time system, it would be expected that the error in the PC-derived data would likely be worse. The error of the PVT-192 is claimed by the manufacturer to be ±1 ms (personal communication; May 5, 2010). Based on the analysis of the baseline sessions of sleep-satiated subjects (Rupp, Wesensten, & Balkin, 2012), which showed an intrasubject variability of 29 ms, we hypothesized that any error of  ≤ 10 ms in the PVT data would be acceptable and well below the threshold of operational/clinical significance (Belenky et al., 2003; Van Dongen, Maislin, Mullington, & Dinges, 2003). Thus, this was the benchmark against which our platform was assessed. Accordingly, we designed a set of experiments to characterize and compare the performance of the two systems, with the ultimate goal of determining whether the PC-PVT platform can be a viable replacement for the PVT-192.

## Method

### System description

The PC-PVT software consists of two logically separate applications, the “Manager” and the “Tester.” The Manager is used by the investigator to create and configure testing protocols, enter subject information, and view the collected data and analysis results. The Tester is used by the subject to perform a 5- or 10-min PVT session.

Our development goal for the Tester was to duplicate the functionality of the PVT-192 as closely as possible. The Tester uses a five-digit millisecond counter presented on the computer screen as the visual stimulus. Instead of the button press implemented in the PVT-192, a mouse button click serves as the response. The PVT protocol requires each stimulus to be delayed by a random period of time (usually between 2 and 10 s), which is referred to as the interstimulus interval (ISI). A response during this period is reported as a “false start” condition. Failure to respond within 65 s of the stimulus is reported as a “no-response” condition. After each valid response, the RT is shown on the screen for 500 ms, the screen is erased, and the execution goes into the next random ISI. This implementation matches the operation of the PVT-192.

The core component of our testing software is written in C and requires Microsoft Windows XP or later to run, with Windows 7 being the preferred OS, due to better support for modern timing hardware (e.g., the High Precision Event Timer). To reduce delays in RT measurements, the application raises its own priority level to have the OS dedicate as much of the central processing unit (CPU) time as possible to the PVT session. The Windows application programming interface (API) QueryPerformanceCounter function is used to assign time stamps of submillisecond precision to key protocol events. The Direct3D API is used to gain access to hardware video acceleration. This provides the application with additional control over how and when the stimulus is presented, and allows it to track the monitor’s refresh cycle to estimate the time when the stimulus actually appears on the screen. The raw-input API is used to detect mouse clicks, which minimizes delays typically associated with mouse input processing by the OS. Finally, a USB gaming mouse with support for 1000-Hz scanning frequency is used, which lowers the error associated with the input hardware from 8 ms (125 Hz default scan frequency) to 1 ms.

At the end of each PVT testing session, RT data are automatically saved to a subject-specific directory in a text-based file format. A copy of the data is also saved in a format used by the PVT-192, making it possible to continue using existing tools (e.g., REACT software) to analyze data collected with the PC-PVT. Once all dialog windows are closed, the application continues running in the background to execute near-real-time individualized analysis algorithms on the most recently obtained data. The algorithm output is saved to the same subject-specific directory as the session data.

**Fig. 1:**
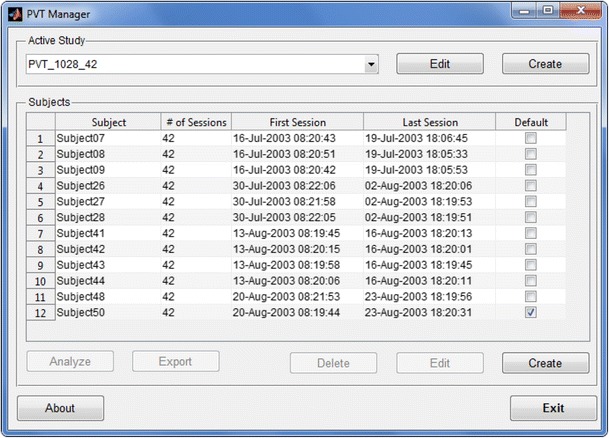
The main PVT Manager window displays the current study name and the list of subjects defined for the study. The session information columns provide an overview of the progress that each subject is making through the study protocol. The buttons below the Subjects list are enabled when a subject is selected and allow for the analysis and export of PVT data and for the management of subject parameters (e.g., subject identification number, left/right hand preference, etc.)

The Manager provides a simple graphical user interface for generating study protocols, exporting data, and visualizing PVT data and predictions. Upon startup, the Manager displays a menu for creating new protocols and configuring the subject information within the current study (Fig. 1). In addition, the investigator may select one or more subjects and export their PVT data and computed summary statistics as a CSV file for offline analysis.

**Fig. 2:**
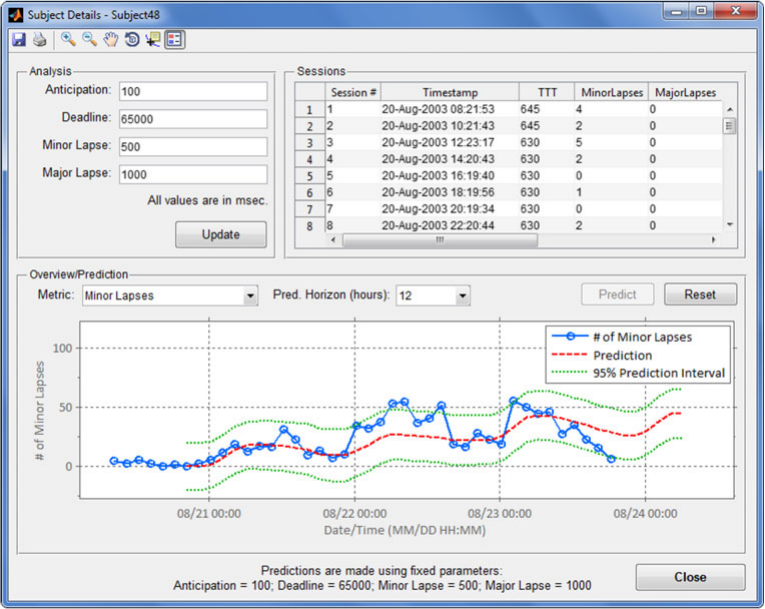
The Subject Details window displays session data and predictions for a single subject. The Analysis panel (top left) is used to configure dependent parameters. The Sessions panel (top right) lists each completed PVT session for the given subject, including the time stamp, configuration parameters, and computed statistics. The Overview/Prediction panel (bottom) displays a plot of the currently selected summary statistics (in the example shown, minor lapses, as defined in the Analysis panel) and outputs from the prediction algorithm (in this case, for a 12-h-ahead prediction). TTT: total trial time, in seconds

Double-clicking a subject ID displays the analysis window (Fig. 2), which is used to view information for all PVT sessions taken by that subject and to plot the raw data, summary statistics, and output of the prediction algorithm. Currently supported summary statistics include: major and minor lapses (which are defined in the Analysis window; see Fig. 2), mean RT, speed, mean of the fastest and slowest 10 % of RTs, and RT divergence (Rajaraman et al., 2012). This window allows for a quick overview of a given subject’s performance across time. The bottom panel of Fig. 2 shows a plot of minor lapses (RTs > 500 ms), individualized predictions, and 95 % prediction intervals for a subject who underwent 85 h of total sleep deprivation. For this example, the prediction algorithms were executed in a post-hoc fashion on an existing dataset.

### Individualized prediction algorithms

The design of PC-PVT provides the means to integrate existing analysis methods, such as our previously reported individualized prediction model (Rajaraman et al., 2008, 2009), directly into the testing module. Whereas before, these algorithms could only be used in a post-hoc fashion, requiring substantial manual effort in obtaining and organizing the data, we can now execute them immediately after the completion of each PVT session. The software provides the means to access not only the most recently acquired RTs but also those from all previous sessions performed by the subject. Once the analysis is completed, algorithm results are stored on the disk along with the session data, which allows researchers to track changes in algorithm output as more data become available.

Our prediction algorithm considers all previously measured performance data for a given subject to customize the model parameters (i.e., to learn the subject’s sleep-loss phenotype) and predict performance up to 24 h into the future. Using a Bayesian approach, which combines a priori information with the measured performance data, the prediction algorithm starts customizing the model for the subject as soon as the first PVT session is completed. As the subject completes additional test sessions, the algorithm starts reducing the weight assigned to the a priori information (here obtained from a fixed, group-average prediction model) and increasing the weight assigned to the measured performance data, as it customizes the model to the specific subject.

### Testing and validation

The goal of our laboratory testing was to determine the extent to which RTs from the PVT-192 match those produced by the PC-PVT. Two main factors account for the degradation in the quality of RT measurements in the PC-PVT. The first relates to the decreased accuracy in the recorded time stamps for the stimulus onset and subject response, due to the multitasking nature of the PC. The second relates to a systematic delay in response detection introduced by the hardware and/or software, which causes a right shift in the RT data. A characterization of these sources of error, which ultimately needs to be accurate to within a few milliseconds, should be devoid of potential confounders associated with intra-subject variability, time-on-task effects (Doran, Van Dongen, & Dinges, 2001), and performance differences due to changes in the human-device interface.

To this end, our solution was to use a separate device capable of detecting the onset of the stimulus and measuring RT with submillisecond precision. One such device, called RTbox, was developed at the University of Southern California (Los Angeles, CA; Li et al., 2010). The RTbox has a built-in light sensor that detects changes in brightness (e.g., the appearance of the millisecond counter on the PC screen or the LED dot-matrix display of the PVT-192, indicating the stimulus onset). A simple two-wire circuit is attached to the response button (left mouse button for the PC, right button for the PVT-192), such that the circuit is closed at the exact moment of the button click. The RTbox contains its own timer, which time-stamps events from the light and button circuits with an accuracy of 0.1 ms (Li et al., 2010). The event code and associated time stamp are transmitted over a USB cable to another PC, which subtracts the time stamp of the light signal from the time stamp of the button click to estimate the “true” RT. A human operator is still required to initiate a response after recognizing the stimulus, but the absolute RT value is irrelevant. Of relevance is the difference between the RTs measured by the RTbox (the true value) and the RTs measured by the device under evaluation (PVT-192 or PC-PVT).

The testing of the PC-PVT was conducted on six different computers (four desktops and two laptops), with production years ranging from 2006 to 2012. We tested both low- and high-end systems, using graphics chips from three major manufacturers (NVIDIA, AMD/ATI, and Intel), to cover the range of hardware that is likely to be used in actual neurobehavioral performance studies. Since the effects of mouse selection on response timing have been previously shown to be significant (Plant, Hammond, & Whitehouse, 2003), two different mouse types were tested to characterize the difference between gaming and non-gaming hardware. The former was Razer Abyssus (Razer USA Ltd., San Diego, CA), designed specifically to reduce lag in fast-paced computer games, and the latter consisted of two standard laser mice from Dell (Round Rock, TX), which typically accompany new desktops. It should be noted that with the exception of the mice, none of the other components were specially selected to reduce the delays inherent in the PC. Using a cathode ray tube (CRT) monitor, for example, instead of the liquid crystal display (LCD), would have likely improved our results due to known delays in the operation of LCD panels. However, because CRTs are becoming much less ubiquitous, such a configuration would not have been representative of the conditions under which our software is likely to be used. Table 1 summarizes the hardware and software configurations for all tested systems, which were all running Microsoft’s Windows 7 Enterprise OS.

**Table 1:** PC configurations used for testing the PC-PVT

PC	Year	CPU	GPU	Monitor	OS
Dell Dimension 5150	2006	Pentium D 820	Radeon X300 SE	Dell 2007FP	64-bit
Dell Dimension 9150	2006	Pentium D 920	Radeon X300 SE	Dell 2007FP	64-bit
Dell OptiPlex 745	2007	Core 2 E6600	Radeon X1300	Dell 2007FP	64-bit
Dell OptiPlex 755	2009	Core 2 E8400	Radeon HD 2400 XT	Dell 2007FP	64-bit
Dell Precision M4500	2010	Core i5-540 M	Quadro FX 880 M	Built-in LCD	32-bit
Lenovo X230	2012	Core i5-3360 M	Intel HD 4000	Built-in LCD	64-bit

PC = personal computer; CPU = central processing unit; GPU = graphics processing unit; OS = operating system (Windows 7 Enterprise)

## Results

**Fig. 3:**
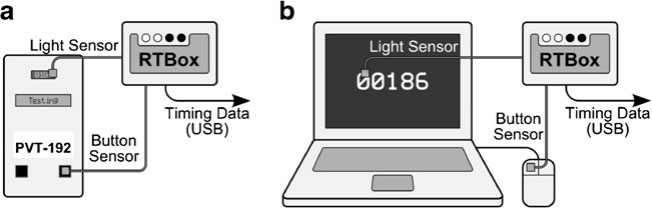
Diagrams of the connection between the RTbox and the device being tested. (A) For the PVT-192, a light sensor was attached from the RTbox to its light-emitting-diode dot-matrix display, and a button sensor was attached from the RTbox to the response button so that both would be triggered at exactly the same time. Events from the two sensors were time-stamped by the RTbox and transmitted via a USB cable to another PC. (B) Similar connections were established in order to characterize the PC-PVT. Reaction times recorded by each device were compared with those derived from the RTbox time stamps

Figure 3 shows a diagram of how the RTbox was connected to the PVT-192 (A) and each of the PCs (B), creating a “closed loop,” meaning a testing setup not dependent on human factors, between the stimulus and the response. A separate PC (not shown) was used to record the output from the RTbox via a USB cable.

We conducted three types of experiments in which we compared the RTBox against (1) the PVT-192, (2) the PC-PVT with a gaming mouse, and (3) the PC-PVT with a standard mouse. Each experiment consisted of 25 min of data collection (five sessions of 5 min each) per device, during which an operator artificially varied the RT from the fastest possible (~160 ms) to ~2,000 ms, in order to cover a wide range of outputs. At the end of each experiment, RTs recorded by the RTbox were subtracted from those recorded by the device being tested. The difference is a measure of the delay associated with the device relative to the RTbox. We used Bland–Altman plots to illustrate the delay and variability of the collected data (Bland & Altman, 1986).

**Fig. 4:**
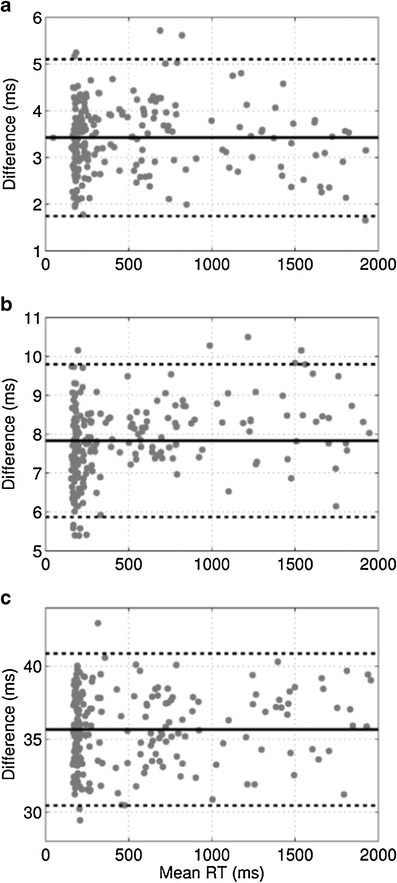
Bland–Altman plots displaying differences in the recorded reaction times (RTs) between the RTbox (reference) and the device being tested. Solid lines represent the means of the differences, and dashed lines represent means ±2 standard deviations (*SD*s). (A) RTbox versus PVT-192 (mean = 3.4 ms, *SD* = 0.8 ms). (B) RTbox versus PC-PVT on a Dell Precision M4500 with a gaming mouse (mean = 7.8 ms, *SD* = 1.0 ms). (C) RTbox versus PC-PVT on a Dell Precision M4500 with a standard mouse (mean = 35.7 ms, *SD* = 2.6 ms). All three plots were cropped at 2,000 ms on the horizontal axis

Figure 4 shows Bland–Altman plots for the three experiments with PC-PVT running on the Dell Precision M4500 laptop. The RTs measured by the RTbox were always less than those recorded by each device, resulting in a positive difference.

Table 2 shows descriptive statistics for the three experiments and all six desktops and laptops, sorted in ascending order by the mean delay relative to the RTbox. With the data obtained from the RTbox taken as the reference (or true) RT, we observe that all devices exhibited measurement delay, with the PVT-192 producing the smallest mean and maximum differences, and the smallest standard deviation. The PC-PVT with the gaming mouse on all six PCs produced the next smallest minimum, mean, and maximum differences, whereas the PC-PVT with the two standard mice yielded the worst performance.

**Table 2:** Reaction time differences (in milliseconds) from the RTbox, sorted by mean time in ascending order

Device	# of RTs	Min	Mean	Max	*SD*
PVT-192	211	1.7	3.4	7.6	0.8
Lenovo X230 (G)	211	1.8	5.3	9.1	1.6
Dell Dimension 9150 (G)	208	1.6	7.8	13.4	2.1
Dell Precision M4500 (G)	211	5.4	7.8	10.5	1.0
Dell Dimension 5150 (G)	213	2.9	7.9	15.2	1.9
Dell OptiPlex 745 (G)	214	2.2	9.1	14.0	2.2
Dell OptiPlex 755 (G)	214	4.9	9.3	14.0	1.6
Lenovo X230 (S1)	213	21.8	30.8	38.7	3.1
Dell Precision M4500 (S2)	213	29.4	35.7	42.9	2.6

G = gaming mouse; S1 = standard mouse 1; S2 = standard mouse 2

## Discussion

The PC-PVT testing results indicate that the hardware component with the most significant impact on the RT data quality is the mouse. When a gaming mouse with the appropriate OS driver was used, all six PCs were able to measure RTs with an average error of less than 10 ms, which is comparable to the PVT-192. The computer age, processor, memory, OS version (32- or 64-bit), and video hardware had a negligible impact (≤4 ms, on average) on the results. In fact, the two old Dell Dimension desktops performed just as well as the much newer and more powerful Dell Precision M4500 laptop. This is not entirely surprising given that the performance of a PC is typically measured in terms of work accomplished in a certain unit of time (throughput), whereas the PC-PVT benefits most from low latency. Thus, certain hardware components and device drivers will be inherently better than others, but this is not a relationship that can be easily deduced simply by looking at the hardware specifications or manufacturing date. In the end, the choice of the input device (i.e., the mouse) is much more significant. With the mouse being the only component changed between Experiments 2 and 3, we expect similar results to be obtained from any system that meets the minimum system requirements (see PC-PVT User’s Guide), which are based on the minimum requirements for running MATLAB. Before each test session, the PC-PVT software verifies the availability of the required hardware video acceleration capabilities and terminates the session with an error if minimum requirements are not met.

The average delay of 7.9 ms for the PC-PVT across all six PC configurations with the gaming mouse constitutes an error of ~3 %, if one assumes an average RT of 240 ms (*SD* = 29 ms) in sleep-satiated individuals (Rupp et al., 2012). This compares to an error of ~1 % with the PVT-192. Thus, for laboratory or clinical studies, the error associated with our system is similar to that of the PVT-192 and is significantly smaller than the intrasubject RT variability of ~29 ms (Rupp, Wesensten, & Balkin, 2012). Most investigators do not implement analysis methods based on the entire raw RT distribution (such as the method described in Rajaraman et al., 2012), but instead use summary statistics extracted from the RT distribution, such as the number of lapses exceeding 500 ms. When these summary statistics are used, the small relative margin of error associated with our system becomes even less relevant.

Of note, the 3.4-ms average delay associated with the PVT-192 is greater than the manufacturer-stated 1 ms (personal communication; May 5, 2010). The difference between our recorded delay and that reported by the manufacturer could be due to the non-instantaneous nature of the light detection circuit, or it could reflect the true delay associated with the PVT-192. Our experimental setup does not allow us to distinguish one from the other.

Because the USB poll rate difference between gaming and standard mice is only 7 ms, the maximum delay of 42.9 ms that we found for one of the standard mice was unexpected. This finding suggests that other hardware design factors can impact the overall data quality. The additional delay is likely caused either by the click detection logic inside the mouse itself or the device driver responsible for reporting mouse events to the OS. Whatever the cause, non-gaming mice potentially introduce an unacceptable amount of delay into the measurements and thus should be avoided. It is worth noting that the RT variability in standard mice was only slightly worse than that of the gaming mouse, but this is device-specific and could be different for other hardware. To be absolutely certain of the timing errors inherent in any specific hardware/software configuration, this testing procedure would need to be repeated using the RTbox, or a similar hardware platform with known characteristics, as the reference.

## Conclusion

We have developed a platform that can be used to conduct simple visual RT testing on a PC. The results of a comparison of this platform with the PVT-192, the current gold standard, revealed only small differences in the quality of the data (in terms of mean RT offset and variability) obtained between the two platforms. These findings indicate that the use of PC-PVT in neurobehavioral performance studies will result in data that are as reliable as those obtained using the hand-held (PVT-192) device.

In addition, we established a verification and validation protocol that can be used to characterize the reliability of almost any device designed for visual RT data collection. The use of the RTbox allowed us to test the system components in a “closed loop”—that is, a context in which the results were independent of human operation—and thus to account for delays introduced only by the stimulus presentation, response detection, and data-processing components.

This platform integrates individualized prediction models directly into the testing software, allowing the model to learn the sleep-loss phenotype of each subject in order to customize and improve the accuracy of predictions. The platform may eventually be ported to smartphones or other portable devices, possibly with the help of hardware implementations (e.g., field-programmable gate arrays) to overcome the limitations in computational power, providing a powerful tool for near-real-time individualized neurobehavioral performance prediction.

## Software availability

The PC-PVT software is freely available and can be downloaded from http://bhsai.org/downloads/pc-pvt/.
